# Impact of regular televisits on unplanned hospital admissions of nursing home residents in rural Germany: a pre-post intervention study

**DOI:** 10.1186/s12877-025-06244-6

**Published:** 2025-09-08

**Authors:** Anne-Catherine Redeker, Tobias Martin, Sarah Veldeman, Carina Barbosa Pereira, Janosch Kunczik, Michael Czaplik, Andreas Follmann

**Affiliations:** 1 Faculty of Medicine, RWTH Aachen University, Pauwelsstraße 30, 52074 Aachen, Germany; 2 Docs In Clouds Telecare GmbH, Vaalser Straße 460, 52074 Aachen, Germany

**Keywords:** Telemedicine, Televisit, Avoidable hospital admission, Ambulatory care-sensitive condition, Nursing home, Nursing home-sensitive condition, General practitioner, Geriatric care.

## Abstract

**Background:**

Hospital admissions occur frequently in nursing homes and are often preventable. Inappropriate hospitalisations due to nursing home-sensitive conditions pose significant risks to residents, place additional strain on emergency departments and hospitals, and thus lead to substantial healthcare costs. In light of demographic changes– characterised by an aging and increasingly multimorbid nursing home population– combined with ubiquitous lack of health care professionals, new strategies are urgently needed to ensure adequate medical care in nursing homes. Telemedicine presents a promising and innovative solution, particularly for rural regions, to improve access to timely medical attention. In this study, we evaluated whether the implementation of regular televisits, in addition to on-site visits, can help decrease unplanned hospitalisations.

**Methods:**

In 2021, a nursing home in rural Germany introduced televisits with a cooperating general practitioner. Data on unplanned hospital admissions was collected for the years 2021/22 and 2018/19, the latter serving as a pre-intervention comparison. Hospital admissions were then compared between the two time periods, as well as between residents of 2021/22 who did or did not receive regular televisits.

**Results:**

Baseline characteristics were comparable between residents of 2018 and 2021, as well as between residents in the telemedical care and the control group. Unplanned hospital admissions significantly decreased (*P* <.0001) after implementation of regular televisits. Furthermore, a significantly lower (*P* =.04) number of hospital admissions was noted among residents in 2021/22 who received additional regular televisits, compared to the control group of residents that only received regular on-site visits.

**Conclusions:**

Implementing regular televisits in the nursing home setting reduced the number of hospital admissions. This is most likely due to more frequent medical assessment, enabling early detection and timely management of deteriorations. By preventing unnecessary hospital admissions residents were spared the physical and psychological burdens connected with emergency transfers and protected from hospital-associated risks. On top of enhancing quality of care for the residents, televisits implementation in nursing homes can contribute to decrease strain on emergency services and hospitals.

**Trial registration:**

Not applicable, as no health-related intervention, modifying biomedical outcome or health-related measures in patients, took place.

**Supplementary Information:**

The online version contains supplementary material available at 10.1186/s12877-025-06244-6.

## Background

Hospital admissions occur frequently among nursing home (NH) residents in Germany [1, 2] and are a major concern within the German healthcare system. Other European countries and the United States also report a significant number of these hospitalisations [3, 4], with rates more than twice as high as for the age-matched community dwellers [5]. Over the past years there has been an increase of hospital admissions [6], and without timely solutions, this trend will most likely progress.

The German Federal Statistical Office estimates that in 2040 the amount of 67-year-olds and older is going to account for 25–27% of the population, while the percentage of the very old (> 80 years) will continue to rise until 2060 and then lie between 9 and 13% [7]. Likewise, the NH population in Germany is rising in numbers [8, 9], becoming older [10] and including a high number of multimorbid patients [11]. The reason for this shift within the NH population lies in the above-mentioned demographic changes and is further aggravated by other factors. One of which is the current rise of ambulatory home care for the older adult population [8]. Technical developments in the field of information and communication technology and their use in this sector [12] enable people to live independently in their homes for longer. While this may sound like a very positive and desirable outcome, the flip side of it is that people enter the NH later, which means at an older age and therefore most likely with more comorbidities.

Another significant challenge to healthcare provision in the context of NHs is the issue of an ubiquitous lack of health care professionals (HCPs). Personnel shortages concern healthcare systems worldwide and are certainly a major challenge for inpatient geriatric care facilities. At present, the medical care of NH residents is mainly provided by general practitioners (GPs) [13] and on-site nursing staff. Germany is experiencing an increasing gap between the demand and supply of ambulatory physicians [14]. This will cause an approximate unmet need of 20 000 GPs in the very near future [15]. Additionally, the shortage of nursing staff for inpatient care, aggravated by the COVID-19 pandemic, could count up to 307 000 nurses in 2035 [9]. As a dire consequence of this situation, the healthcare of a higher number of NH residents, who are carrying a larger burden of disease, will need to be provided with even fewer personnel resources. This will certainly impact the quality of medical care that can be provided. Especially rural areas, where the accessibility to HCPs is already limited [16], are vulnerable to these developments.

The lack of GPs impacts the medical care of NH residents immensely [17, 18]. With fewer on-site visits clinical signs of a decompensating chronic disease or health status deterioration might be overlooked, leading to an acute event of illness. In which case, nursing staff is often forced to contact emergency services [19]. In fact, a study analysing data from Switzerland showed an increase of ambulance interventions in NHs by 68.9% between 2004 and 2013 [20]. The involved paramedics or emergency physicians, who are not acquainted with the patient’s medical history, tend to take patients to the already crowded emergency departments (EDs) [21, 22], potentially resulting in an inappropriate, avoidable hospital admission [19, 23].

These hospital admissions, which could have been prevented by better ambulatory care, are so called ambulatory care-sensitive conditions (ACSCs) and serve as a quality indicator of ambulatory healthcare [24]. The ACSCs vary between countries due to the different organisation of the healthcare sector, demographical and environmental factors. For Germany, a group of forty physicians selected from all relevant medical disciplines defined a core list of 22 ACSC diagnosis groups [25]. A significant number of hospitalisations in NHs, approximately between 30% and 50%, have been found to be caused by ACSCs [1, 26, 27]. Since NH residents present a geriatric patient group, which differs significantly from the rest of the population, mostly concerning age, care needs, comorbidities, and disease spectrum [1, 28], a group of experts has developed an equivalent list comprising 58 diagnoses of relevant nursing home-sensitive conditions (NHSCs) for which hospitalisations are potentially avoidable [28].

The consequences of NHSCs can be serious and can impact patients’ health status severely. This is especially true for geriatric patients. A hospital stay can be associated with a decline in physical function due to confusion or disorientation [29, 30] and NH residents that are torn out of their daily environment have a high likelihood of manifesting a delirium [31], since it is often associated with pre-existing dementia. Further hospital-associated health complications, such as nosocomial infections, worsen the outcome. In fact, the in-hospital mortality among NH residents is high, reaching up to 34% [3, 5, 31].

Avoiding hospitalisation therefore protects residents from major risks, while care-providers save on effort, time, and resources. Additionally, healthcare insurances benefit from important cost-savings [32, 33]. For 2017, the total amount of potentially avoidable costs, caused by NHSCs in Germany was estimated at around 760 million euros [28].

Telemedicine may help to address many of the challenges that inpatient geriatric care facilities face. Telemedicine has recently experienced a worldwide boom during the COVID-19 pandemic [34–36]. Though certain perceived barriers to the use of telehealth applications may apply to the mostly older residents in NHs (such as lack of knowledge and familiarity with technology) [37], it has been proven to be well accepted among older patients [38–40]. Digital familiarity and perceived usefulness can be mentioned as having a positive impact on acceptance and satisfaction with telemedicine [41, 42]. Previous studies showed that telemedical solutions have the potential to improve quality of care and reduce hospital admissions for NH residents [43–45]. Hofmeyer et al. observed a significant decrease of unplanned hospital transfers from 39 to 17% (2012–2015), while providing a 24/7 teleconsultation service to rural long-term care facilities [46]. Further studies with different models of care, providing unscheduled telemedical assessment, also reported a decline of ED visits and/or reduction in hospital admissions for NH residents [47–51]. Besides acute telecare, routine scheduled teleconsultations conducted by different specialists (including geriatricians) were associated with fewer transfers and a lower hospitalisation rate as well [33, 52, 53]. Telemedical consultation reduced the hospitalisation of NH residents in 14 out of 16 studies (published between 2001 and 2022) identified in a review by Valk-Draad & Bohnet-Joschko [54].

While previous studies have primarily focused on the use of telemedicine for acute, unplanned assessments - often involving various specialists responding to sudden health deteriorations - this study investigates a different approach. Due to the continuous, longstanding care of GPs in NHs, the GPs alongside with the on-site nursing staff, are potentially best suited to detect and asses health status deterioration early on. Since GPs also take over a major part of chronic disease management, intensified follow-up by GPs through televisits could prevent a relevant amount of NHSCs and potentially avoid hospitalisation. We examined the effect of scheduled, regular televisits conducted by a GP on inpatient nursing home care. The aim was to evaluate whether these televisits, provided in addition to existing on-site visits, can reduce unplanned hospital admissions for NH residents.

## Methods

### Study design

**Fig. 1:**
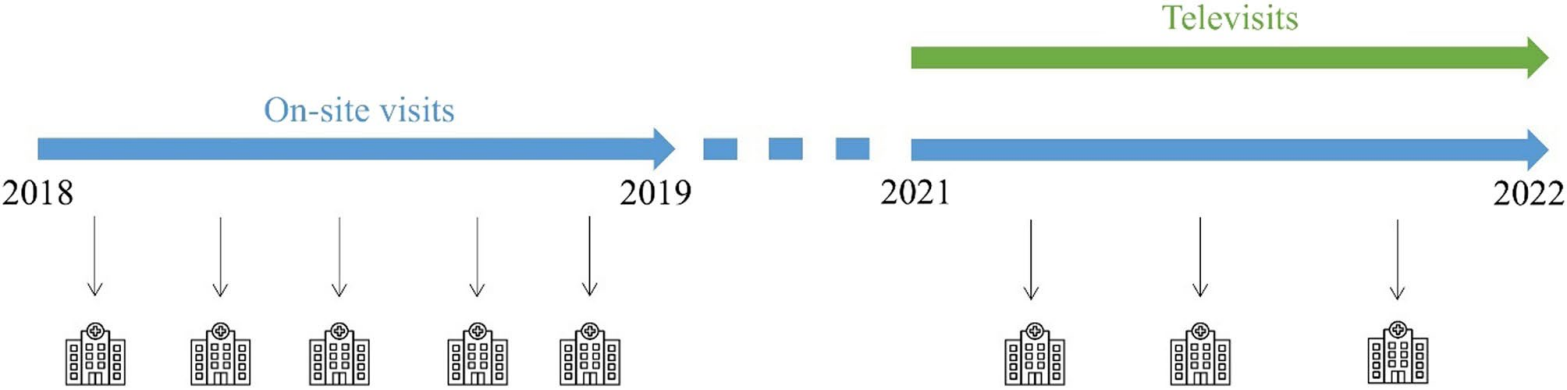
The study design, showing the pre-intervention period 2018/19 and the post-intervention period 2021/22 with regular televisits complementing on-site NH visits. The hospital symbols represent unplanned hospital admissions of NH residents. The NH population of 2018/19 only received on-site visits, while the NH population of 2021/22 was split into the telemedical care group, receiving regular televisits in addition to on-site visits, and the control group only receiving on-site visits

In order to evaluate the effect of regular televisits in inpatient geriatric care, we conducted a single-centre, retrospective, longitudinal, pre-post intervention study (Fig. 1). In 2021, a NH in rural Germany introduced televisits, connecting the facility to one of its cooperating GPs (referred to as GP1). Over a period of 12 months, from the 01.08.2021 to the 31.07.2022, GP1 provided care for an average of 27 residents (32% of the NH population), by conducting regular televisits, in addition to on-site NH visits. These residents formed the telemedical care group. The remaining 57 residents (68%) were cared for by other GPs who did not offer telemedical care and thus served as the concurrent control group. To evaluate the effect of televisits, data from an earlier 12-month period (01.08.2018 to the 31.07.2019), prior to the introduction of televisits, served as a pre-intervention comparison. Unplanned hospital admissions from the NH residents were analysed and compared before (2018/19) and after (2021/22) intervention, meaning since the introduction of televisits. A sub-analysis compared the hospitalisations between the telemedical care group and the control group within the 2021/22 time period. Baseline characteristics of the NH populations were collected for both reference dates: 01.08.2018 and 01.08.2021, in order to compare the populations (2018/19 and 2021/22) and both groups of the 2021/22 NH population (telemedical care and control group). All residents aged ≥ 18 years who received long-term inpatient care in the NH were included.

### Setting

The study took place in a NH within the rural district Euskirchen in North Rhine-Westphalia, Germany (Stiftung Evangelisches Alten- und Pflegeheim Gemünd). The population density there is 158 inhabitants per km² [55] and the GP coverage rate is 109.8%, meaning that there is neither an undersupply nor oversupply of GPs. However, only 75.8% of patients are cared for by a GP located less than 10 km away [56].

The NH accommodates a total of 84 residents in four different residential areas. GP1 oversees approximately one-third of the residents, the other residents in the NH are followed up by other GPs. Before introduction of televisits, GP1 conducted on-site visits every second week. This was maintained during 2021/22 and complemented by regular televisits every second week whenever GP1 did not visit on-site. In weekly meetings between GP1 and the on-site nursing staff, residents were discussed and identified as candidates for televisits either to follow-up on an ongoing health issue or to assess a recent deterioration in their condition. The decision to conduct a televisit was made solely at GP1’s discretion, based on his professional medical judgment. As a result, not all residents of the telemedical care group received televisits every two weeks. During fixed televisit hours, scheduled biweekly by GP1 in coordination with the nursing staff, a varying number of residents identified as needing consultation were assessed via televisit. The content and scope of each televisit varied depending on the resident’s clinical needs, ranging from simple discussions of health status and treatment plans to targeted examinations using point-of-care (POC) diagnostic devices. There was no standardised protocol guiding these assessments. Importantly, the televisits served solely as a complementary tool and never replaced on-site visits. The entire nursing staff was educated and trained through continuous telemedical exercise courses that spanned the duration of the post-intervention period (2021/22).

The other GPs, responsible for the remaining residents in the control group, continued treating their patients by regular on-site visits only, following their respective schedules. In unforeseen acute situations, the treating GPs (GP1 and other GPs) conducted unscheduled on-site visits whenever they were reachable and not prevented by conflicting duties (e.g. simultaneous practice hours). In cases assessed as life-threatening, emergency services were contacted.

While the resident distribution always stayed about one-third for GP1 and two-thirds for other GPs, group composition slightly changed over time, because residents dropped out (for example (e.g.), by death) and were added (e.g., moving-in) to the two different groups.

### Televisits

**Fig. 2:**
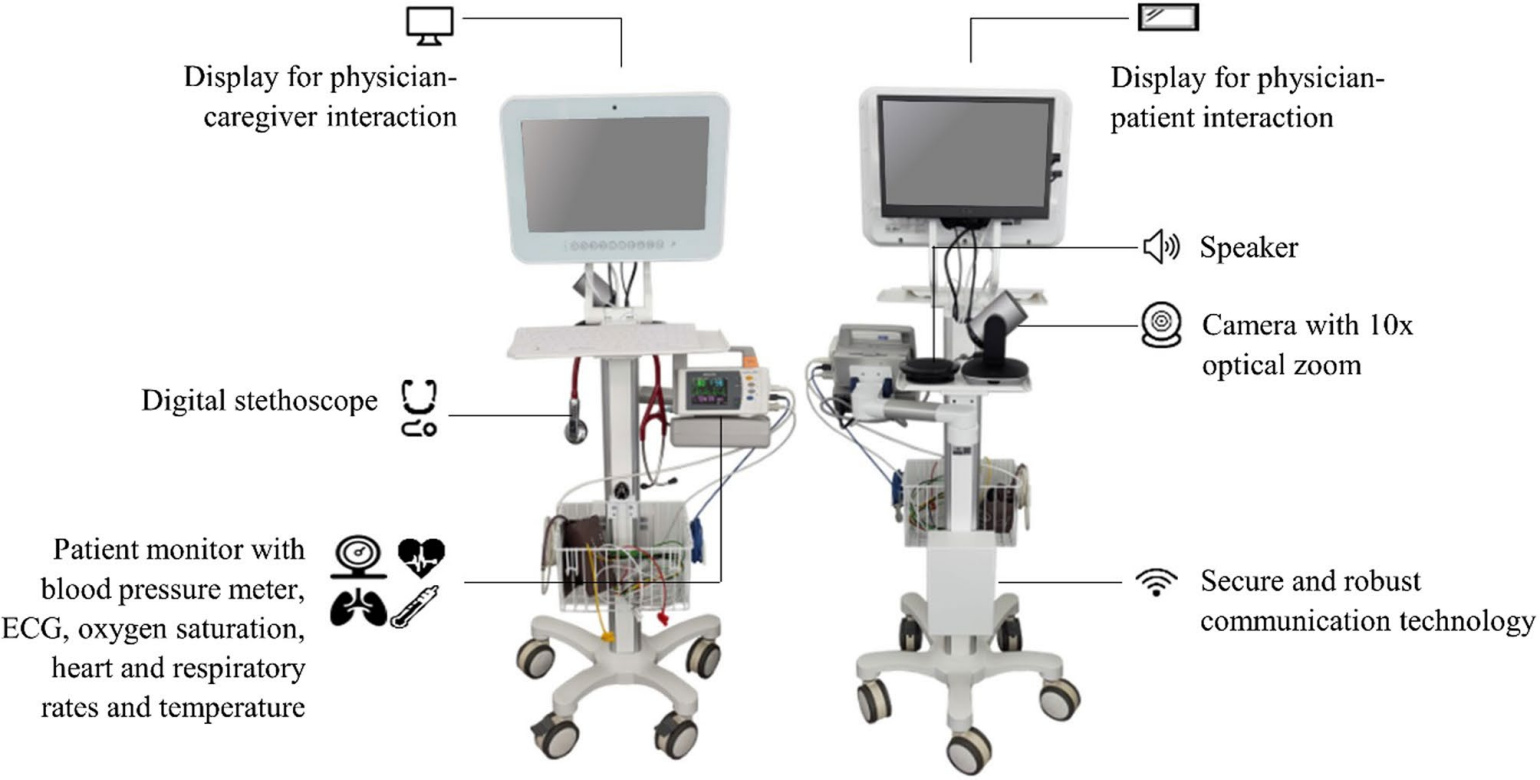
The commercially available “TeleDoc Mobile” cart system equipped with medical devices for POC diagnostic, which is shown from both sides

All televisits were performed using the commercially available “TeleDoc Mobile”, a medical cart system (Docs in Clouds TeleCare GmbH, Aachen, Germany) (Fig. 2). The “TeleDoc Mobile” system had already been tested for inpatient geriatric care within a prior pilot study of our research group, showing technical feasibility of televisits and acceptance of the system [57]. In addition to audio and video communication between patient and physician (definition of a video consultation), a televisit allows diagnostic procedures by on-site nursing staff, to whom therapeutic measures can be delegated. The currently available “TeleDoc Mobile” cart is equipped with displays, secure communication technology and medical devices for POC diagnostic such as a digital stethoscope (Littmann stethoscope model 3200; 3 M), a blood pressure meter (BU 540 connect; medisana GmbH), a blood glucose meter (MediTouch 2; medisana GmbH), an electrocardiogram (ECG; WIWE pocket ECG; myWIWE Diagnosztika Kft), and an adjustable room camera with optical zoom (PTZ Pro 2; Logitech International S. A.).

Via the installed “TeleDoc Mobile” software, a televisit could be started by the HCPs (GP or nursing staff), connecting GP1 to the patient and nursing staff in the NH. The visit took place using audio- and video communication, allowing interaction between the patient, GP1 and nurse. If requested by GP1, the nursing staff used the integrated devices for asynchronous on-site medical examination, meaning that vital parameters were being taken before the televisit was started. This modality of asynchronous televisits used a web-based waiting room for the GP, a physical waiting room in the NH and a defined televisit room, described in further detail in the article of Martin et al. [58]. The gathered information from anamnesis and POC diagnostic subsequently served as basis for further decision-making to GP1. If necessary, GP 1 could subsequently delegate the implementation of specific therapeutic measures to the on-site nurse. During televisits, data safety was guaranteed through end-to-end encryption for data transmission, as well as for bidirectional audio- and video connection. The “TeleDoc Mobile” software also allowed asynchronous communication between GP1 and the nursing staff. Documents could be exchanged and uploaded to the televisit record without being in an active televisit. Particularly, the nursing staff on-site could prepare for the televisits by documenting diagnostic data, considered relevant to the individual case. This data could then also be viewed by GP1 prior to the televisit.

Several kick-off training sessions for the NH nursing staff and the cooperating GP were held in small groups to introduce the medical “TeleDoc Mobile” system before the beginning of the study. The practical testing of the medical devices for POC diagnostic was a central component of the initial training concept. Thereafter, nursing staff trainings took place, improving the handling with the “TeleDoc Mobile” software and the medical devices, while reflecting the importance of these devices for their work and the medical care of NH residents. Simulated scenarios were used to train and consolidate the acquired skills. The training courses took place in person, also some training units could be completed online. The article of Martin et al. shows a timeline of the telemedical implementation process including further information regarding the training [58].

### Data collection

Data was collected retrospectively by accessing the NH documentation software, using specific lists generated by the software, as well as the medical care documentation record. The following baseline population data, presented in Tables 1 and 2, were documented systematically for every resident on the 01.08.2018 and 01.08.2021: general personal characteristics (including care level), length of stay in the NH and occurred hospitalisations, geriatric, cardiovascular, ischaemic, neuropsychiatric, respiratory, and gastrointestinal pre-existing conditions, as well as several non-classified diagnoses. Furthermore, the following information concerning all unplanned hospital admissions of residents occurring in both time periods was retrieved: time and type of transfer, hospitalisation cause (medically confirmed reason for hospital admission, determined by the responsible GP, the emergency paramedics, or the emergency physician) which was classified into specific groups (e.g., gastrointestinal problems, fall or cardiovascular disorder), in-hospital diagnosis, length of stay, event of death and the current treating GP of the resident. The number of NH residents in long-term inpatient care and the treating GP of each resident was retrieved for the six following key dates: 01.08.2018, 01.02.2019, 31.07.2019, 01.08.2021, 01.02.2022 and 31.07.2022, in order to calculate the average number of residents for the time period of 2018/19 and 2021/22, as well as for the different GP groups (GP1 and other GPs).

**Table 1:** Baseline characteristics of the NH population on the 01.08.2018 and the 01.08.2021: data listed as mean with standard deviation (SD) and counts (n) with percentages (%)

		Missing	01.08.2018	01.08.2021	*P*-Value
n			85	84	
Gender, n (%)	Male	0	21 (24.7)	15 (17.9)	0.368
	Female		64 (75.3)	69 (82.1)	
Age, mean (SD)		0	86.1 (6.9)	85.7 (8.3)	0.753
Care level, n (%)	2	0	10 (11.8)	6 (7.1)	0.720
	3		24 (28.2)	26 (31.0)	
	4		29 (34.1)	27 (32.1)	
	5		22 (25.9)	25 (29.8)	
GP, n (%)	GP1	0	23 (27.1)	23 (27.4)	1.000
	Other GPs		62 (72.9)	61 (72.6)	
Length of stay in the NH, mean (SD)		0	2.8 (3.3)	2.8 (3.6)	0.871
Number of hospitalisations since moving into the NH, mean (SD)		0	1.8 (2.3)	1.5 (2.6)	0.341
Days of hospitalisation since moving into the NH, mean (SD)		0	16.4 (24.4)	13.2 (23.7)	0.392
Geriatric syndromes:					
Aconuresis, n (%)		0	31 (36.5)	39 (46.4)	0.247
Anal incontinence, n (%)		0	28 (32.9)	26 (31.0)	0.911
Dementia/Cognitive impairment, n (%)		0	48 (56.5)	53 (63.1)	0.471
Fall risk, n (%)		1	75 (88.2)	65 (78.3)	0.129
Gait & mobility disorder, n (%)		0	15 (17.6)	17 (20.2)	0.815
Immobility, n (%)		0	6 (7.1)	6 (7.1)	1.000
Cardiovascular risk factors:					
Arterial hypertension, n (%)		0	58 (68.2)	67 (79.8)	0.126
Diabetes mellitus, n (%)		0	22 (25.9)	24 (28.6)	0.826
Adiposity (BMI≥30), n (%)		1	16 (18.8)	14 (16.9)	0.897
Hyperlipidaemia/Dyslipidaemia, n (%)		0	44 (51.8)	50 (59.5)	0.390
MARKER ischaemic risk:					
CHD/Status post cardiac infarction/Atherosclerosis/Vascular stenosis/(P)AOD, n (%)		0	39 (45.9)	38 (45.2)	1.000
MARKER neuropsychiatric risk:					
Dementia/Cognitive impairment/Schizophrenic disorder/Psychotic disorder, n (%)		0	50 (58.8)	53 (63.1)	0.681
MARKER respiratory risk:					
COPD/Bronchial asthma/Chronic bronchitis, n (%)		0	7 (8.2)	11 (13.1)	0.439
MARKER gastrointestinal risk:					
Gastritis/Oesophagitis/Gastroesophageal reflux, n (%)		0	21 (24.7)	19 (22.6)	0.890
Cardiac arrhythmia, n (%)		0	29 (34.1)	37 (44.0)	0.244
Cardiac pacemaker, n (%)		0	9 (10.6)	8 (9.5)	1.000
Cardiac insufficiency, n (%)		0	13 (15.3)	21 (25.0)	0.167
Renal insufficiency, n (%)		0	12 (14.1)	24 (28.6)	0.035
Hypotension, n (%)		0	1 (1.2)	1 (1.2)	1.000
Hypothyroidism, n (%)		0	8 (9.4)	8 (9.5)	1.000
Chronic pain, n (%)		0	11 (12.9)	11 (13.1)	1.000
Morbus Parkinson/Parkinson syndrome, n (%)		0	10 (11.8)	4 (4.8)	0.170
Status post apoplexy/TIA, n (%)		0	23 (27.1)	21 (25.0)	0.897

*Abbreviations:NH* Nursing home, *BMI* Body mass index, *CHD* Coronary heart disease, *(P)AOD* (peripheral) arterial occlusive disease, *COPD* Chronic obstructive pulmonary disease, *TIA* Transient ischaemic attack

**Table 2:** Baseline characteristics of the telemedical care group and the control group on the 01.08.2021: data listed as mean with standard deviation (SD) and counts (n) with percentages (%)

		Missing	Telemedical care group	Control group	*P*-Value
n			23	61	
Gender, n (%)	Male	0	6 (26.1)	9 (14.8)	0.337
	Female		17 (73.9)	52 (85.2)	
Age, mean (SD)		0	86.0 (8.2)	85.6 (8.4)	0.876
Care level, n (%)	2	0	2 (8.7)	4 (6.6)	0.886
	3		6 (26.1)	20 (32.8)	
	4		7 (30.4)	20 (32.8)	
	5		8 (34.8)	17 (27.9)	
Length of stay in the NH, mean (SD)		0	3.7 (4.0)	2.4 (3.3)	0.163
Number of hospitalisations since moving into the NH, mean (SD)		0	3.0 (4.1)	0.9 (1.4)	0.022
Days of hospitalisation since moving into the NH, mean (SD)		0	24.7 (32.9)	8.9 (17.5)	0.037
Geriatric syndromes:					
Aconuresis, n (%)		0	12 (52.2)	27 (44.3)	0.687
Anal incontinence, n (%)		0	9 (39.1)	17 (27.9)	0.465
Dementia/Cognitive impairment, n (%)		0	15 (65.2)	38 (62.3)	1.000
Fall risk, n (%)		1	21 (91.3)	44 (73.3)	0.134
Gait & mobility disorder, n (%)		0	6 (26.1)	11 (18.0)	0.543
Immobility, n (%)		0	2 (8.7)	4 (6.6)	0.663
Cardiovascular risk factors:					
Arterial hypertension, n (%)		0	18 (78.3)	49 (80.3)	1.000
Diabetes mellitus, n (%)		0	9 (39.1)	15 (24.6)	0.296
Adiposity (BMI≥30), n (%)		1	4 (18.2)	10 (16.4)	1.000
Hyperlipidaemia/Dyslipidaemia, n (%)		0	13 (56.5)	37 (60.7)	0.924
MARKER ischaemic risk:					
CHD/Status post cardiac infarction/Atherosclerosis/Vascular stenosis/(P)AOD, n (%)		0	8 (34.8)	30 (49.2)	0.349
MARKER neuropsychiatric risk:					
Dementia/Cognitive impairment/Schizophrenic disorder/Psychotic disorder, n (%)		0	15 (65.2)	38 (62.3)	1.000
MARKER respiratory risk:					
COPD/Bronchial asthma/Chronic bronchitis, n (%)		0	3 (13.0)	8 (13.1)	1.000
MARKER gastrointestinal risk:					
Gastritis/Oesophagitis/Gastroesophageal reflux, n (%)		0	9 (39.1)	10 (16.4)	0.054
Cardiac arrhythmia, n (%)		0	10 (43.5)	27 (44.3)	1.000
Cardiac pacemaker, n (%)		0	2 (8.7)	6 (9.8)	1.000
Cardiac insufficiency, n (%)		0	5 (21.7)	16 (26.2)	0.888
Renal insufficiency, n (%)		0	6 (26.1)	18 (29.5)	0.969
Hypotension, n (%)		0	1 (4.3)		0.274
Hypothyroidism, n (%)		0		8 (13.1)	0.100
Chronic pain, n (%)		0	8 (34.8)	3 (4.9)	0.001
Morbus Parkinson/Parkinson syndrome, n (%)		0	1 (4.3)	3 (4.9)	1.000
Status post apoplexy/TIA, n (%)		0	4 (17.4)	17 (27.9)	0.480

*Abbreviations:NH* Nursing home, *BMI* Body mass index, *CHD* Coronary heart disease, *(P)AOD* (peripheral) arterial occlusive disease, *COPD* Chronic obstructive pulmonary disease, *TIA* Transient ischaemic attack

### Statistical methods

The two-sample t-test was applied on continuous variables and the chi-squared or Fisher’s exact test (depending on the number of observations) on categorical variables. Continuous variables were checked for normal distribution before applying the two-sample t-test and are summarised by mean and standard deviation. To compare the total frequency of unplanned hospitalisation between the two time periods and groups, we applied the Fisher’s exact test, which is a nonparametric test, especially for small sample sizes. Hospitalisation was considered as an event that occurred or not (categorical outcome). As we compared proportions of a categorical outcome for independent and not correlated groups, the Fisher’s exact test was used. Solely two-tailed statistical tests were used to calculate the *P*-value, whose statistical significance level was defined by < 0.05. All statistical analysis were performed in Python (version 3.9.7; Python Software Foundation), using the open-source cross-platform integrated development environment (IDE) Spyder (version 5.1.5; Spyder Team and collaborators) and the web-based interactive computational environment Jupyter Notebook (version 3.9.7; Project Jupyter). The population and group comparison tables were produced using the open-source Python package named *tableone* [59]. Missing data concerning baseline characteristics and hospitalisation causes are displayed in the tables and were not imputed for statistical comparison.

## Results

### Study population

Baseline characteristics were assessed for all residents on the key dates 01.08.2018 and 01.08.2021, respectively, and are presented in Table 1. The two populations are comparable, comprising 85 residents in 2018 and 84 residents in 2021. The mean age in both populations was approximately 86 years, with all but one resident (belonging to the population of 2021) aged over 60 years (99.4%), and the majority of residents were female (75.3% in 2018 and 82.1% in 2021, *P* =.368). The care level distribution was similar, with most residents classified in level 4 (34.1% and 32.1%), followed by level 3 and 5, both compromising about 30% of the residents and ending with the lowest care level 2 (*P* =.720). No significant difference was noticed regarding the specific geriatric syndromes, such as aconuresis and (&) anal incontinence, dementia & cognitive impairment, fall risk, gait & mobility disorder, and immobility (see *P*-values in Table 1). The cardiovascular risk factors did not significantly differ between both populations, with arterial hypertension being the most frequent diagnosis (68.2% and 79.8%, *P* =.126). Furthermore, relevant diagnoses for ischaemic, neuropsychiatric, respiratory, and gastrointestinal risk were grouped into specific markers, which were comparable as well. For example, 45.9% of the residents in 2018 and 45.2% in 2021 (*P* = 1.00) had at least one disease associated with increased ischaemic risk (e.g., coronary heart disease or peripheral arterial occlusive disease). For further non-classified diagnoses, renal insufficiency was twice as common among residents in 2021 (28.6%) than in 2018 (14.1%) and the only significant difference between the two populations (*P* =.035). Eventually, the mean length of stay in the facility was 2.8 years for both populations.

In addition, comparing the resident group cared for by GP1 in 2018 and 2021, no significant difference in baseline characteristics was found between the resident group of GP1 in 2018 and 2021 (telemedical care group) [Supplementary Material 1].

Looking at the population for 2021 (data retrieved on the 01.08.2021), the telemedical care group counted 23 residents, whereas the control group included 61 residents (Table 2). Neither the age, gender nor care level distribution was significantly different between the two groups (see *P*-values in Table 2). Same for the specific geriatric syndromes, cardiovascular risk factors and four different markers. For gastrointestinal risk factors, the *P*-value was close to significance level (39.1% in the telemedical care group and 16.4% in the control group, *P* =.054). Significant difference was noted for the three following characteristics: diagnosis of chronic pain (*P* =.001), as well as number (*P* =.022) and days (*P* =.037) of hospitalisation since moving into the NH. In fact, residents in the telemedical care group had had more hospital admissions since moving into the NH (mean 3.0 to mean 0.9) and a higher mean of hospital days since moving into the NH (24.7 to 8.9), than residents in the control group. Both were mainly due to a few statistical outliers, values that lay more than 1.5 times the interquartile range above the upper quartile.

### Hospital admissions

**Fig. 3:**
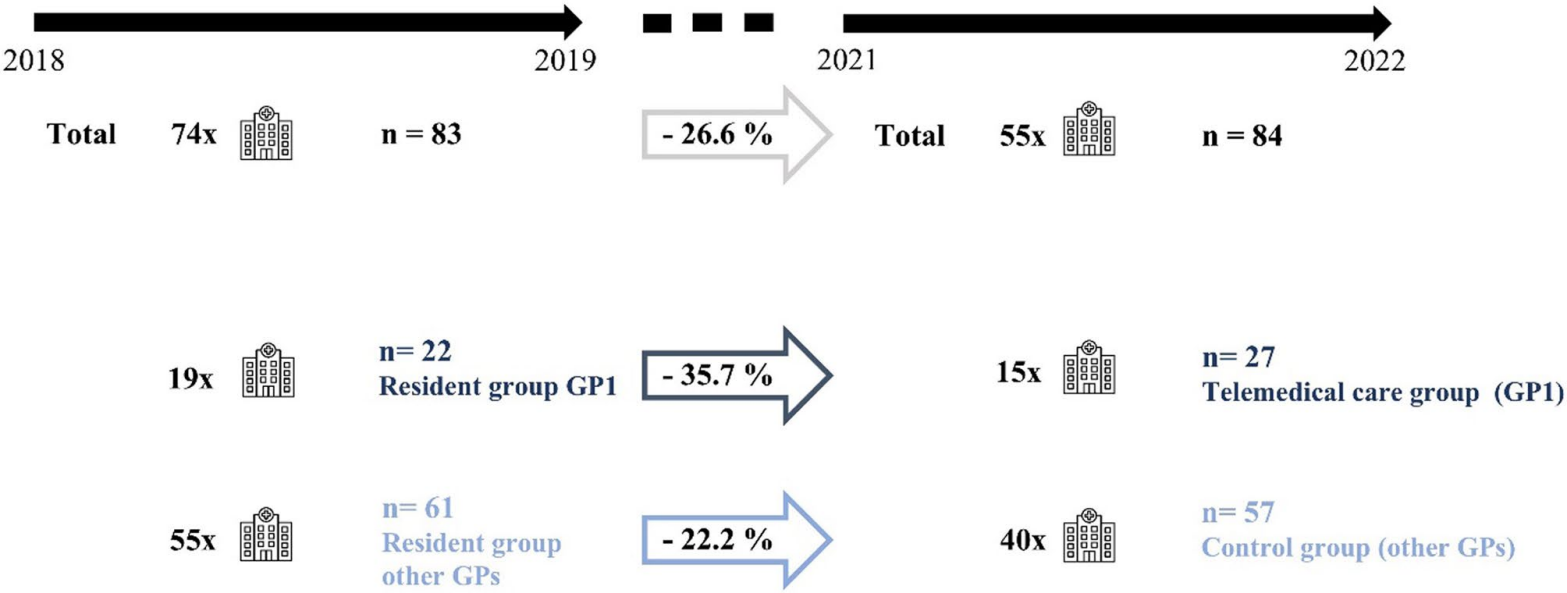
The number of unplanned hospital admissions of NH residents, represented by the hospital symbols, for the pre-intervention period 2018/19 and the post-intervention period 2021/22. The total of unplanned hospital admissions is shown at the top and broken down below into the different GP groups. The average number of NH residents in total and per group is listed with n. The arrows indicate the relative reduction (in percent, %) of admissions in total and per group between 2018/19 and 2021/22

For the time period of 2018/19, 74 unplanned hospitalisations were reported for an average NH occupancy of 83 residents. During 2021/22, 55 unplanned hospital admissions occurred for an average NH occupancy of 84 inpatient care residents. Thus, post-intervention (time period of 2021/22), hospital admissions significantly decreased by a total of 19, corresponding to a relative reduction of 26.6% (*P*<.0001). Of the 55 unplanned hospitalisations in 2021/22, 15 concerned patients receiving regular televisits by GP1. On average, the telemedical care group counted 27 residents. In 2018/19, 19 of the 74 hospital admissions occurred to patients treated by GP1, who cared for an average of 22 residents during that time period. Hence, within the group of GP1, admissions were reduced by 35.7% (*P*<.00001), while for the resident group treated by other GPs, the reduction was by 22.2% (*P*<.001) (Fig. 3).

As mentioned above, 15 of the 55 hospital admissions in 2021/22 concerned residents of the telemedical care group, while the remaining 40 admissions occurred to residents treated by other GPs with regular on-site visits only. On average, the control group counted 57 residents. Thus, during 2021/22, the number of unplanned hospital admissions was significantly lower for residents receiving regular televisits (*P*=.04).

Looking at the comparison time period of 2018/19, there was no significant difference in unplanned hospital admissions between the resident group of GP1 and the resident group treated by other GPs (*P*=.5) (moreover baseline characteristics and hospitalisation causes were comparable for this analysis [Supplementary Materials 2 and 3]).

Following hospitalisation causes were identified for unplanned hospital admissions in 2021/22: breathing difficulties/dyspnoea, gastrointestinal problems, seizure, fall, general health status deterioration (unclear genesis), urologic problems, pain of the lower extremities/thorax/abdomen, hemic anomaly, and syncope. For three hospitalisations, the documentation of cause was missing. For the comparison time period of 2018/19, hospitalisation causes were partly identical to 2021/22, adding: cardiovascular disorder, nephrological problems, suspected apoplexy/transient ischaemic attack, suspected pneumonia, psychopathological anomaly, suspected thrombosis, oedema, inflammation signs of the lower extremities, exsiccosis, dermatological anomaly, medication adjustment and suspected lung embolism. There was no significant difference in distribution of the mentioned causes between admissions of 2021/22 and 2018/19 [Supplementary Material 4].

Comparing the hospitalisation causes of 2021/22 between the telemedical care group and the control group [Supplementary Material 5], only the distribution of general state of health deterioration differed significantly (*P* =.046). The latter was documented ten times for hospitalisations concerning the control group, and not once for unplanned admission within the telemedical care group.

### Televisits

For the study period of 12 months (01.08.2021 to the 31.07.2022), 39 weeks with televisits were recorded. Out of 52 weeks, 6 must be deducted due to vacation of GP1 and during another 7 weeks, televisits could not take place due to other reasons. Hence, a total of 119 televisits occurred over a period of 39 weeks.

## Discussion

### Principal findings

This study represents the first field trial to assess the impact of regular televisits conducted by a GP on unplanned hospital admissions in a NH in rural Germany. The implementation of televisits, connecting the NH to one of its cooperating GPs, was associated with a significant reduction of 26.6% in unplanned hospitalisations for all residents. Interestingly, even residents who did not receive regular televisits, serving as control group, showed a reduction of 22.2% in unplanned hospitalisations. However, the reduction was significantly higher among residents receiving telemedical care, with a decrease in hospital admissions of 35.7%. Furthermore, the number of unplanned hospital admissions was significantly lower in the telemedical care group, compared to the control group. Since there was no significant difference for the number of hospital admissions between the corresponding groups pre-intervention (2018/19), the effect does not seem to be assignable to the connected GP. The few significant differences in baseline characteristics can be considered of small relevance for the analysis. Regarding the difference of renal insufficiency distribution between the NH population of 2018 and 2021, there was no significant difference in hospitalisation cause for nephrological problems (*P* =.511) between 2018/19 and 2021/22 [Supplementary Material 5].

### Interpretation of findings

Our findings strongly suggest that integrating regular televisits into the routine care provided by the GP can effectively reduce unplanned hospital admissions. Several factors are likely to contribute to this outcome.

Firstly, televisits enhanced the frequency and continuity of medical follow-up care. While on-site visits by the GP typically occurred twice a month, the addition of biweekly televisits ensured that residents received medical attention more frequently. This allowed for closer monitoring of chronic health conditions and earlier detection of clinical deteriorations. NHSCs, such as infections or exacerbations of chronic diseases, can often be effectively managed within the facility if identified early. The televisits provided a low-threshold, easily accessible form of medical oversight that facilitated prompt responses to concerns raised by nursing staff or residents, thereby reducing delays in clinical decision-making and interventions.

In this regard the implementation of telemedicine may also contribute to addressing one of the most pressing challenges in healthcare: personnel shortages [9, 15]. With an already critical and growing shortage of GPs– particularly in rural regions– televisits offer a time-efficient and resource-sparing modality for physicians to provide care. By eliminating travel time and enabling remote assessments, GPs can maintain continuity of care for their NH patients more effectively and with less logistical burden. This not only makes the provision of NH care more attractive and sustainable for GPs but may also help to ensure medical care access in areas with limited physician availability.

Nursing staff likewise benefits from this model. Through the ability to address medical concerns directly and routinely with the treating GP, they may experience relief in their day-to-day work. Concerns no longer have to accumulate or wait for the next scheduled visit, and support in decision-making is more readily available.

Another factor contributing to the reduction in hospital admissions in our study appears to be the ongoing training of the nursing staff during the implementation of the televisits. These sessions not only addressed the use of the telemedical platform but also focused on the correct application of diagnostic POC devices. These trainings most likely enhanced staff competence and confidence in performing diagnostics and interpreting clinical findings. Furthermore, the involvement of nursing staff during televisits– such as performing delegated therapeutic measures or diagnostic procedures– shifted their role from passive observers to active participants in medical care. This observation aligns with findings by Sävenstedt et al. [60], who highlighted the evolving roles of nursing staff in telemedical contexts. Additionally, a study by Zúñiga et al. [61] demonstrated that a geriatric nurse-led model of care was effective in reducing unplanned transfers from NHs to hospitals, supporting the notion that expanding the clinical responsibilities of trained nursing home staff can be beneficial. Altogether, this expansion of responsibilities may have strengthened the nursing staff’s clinical judgement, sharpened their awareness of subtle changes in residents’ health status, and deepened their understanding of the potential consequences. These effects on perceived clinical competence, role identity, and professional self-efficacy are further substantiated by the qualitative findings of Martin et al. [58].

It seems likely that these secondary effects of our study which were achieved through above mentioned continuous staff trainings, contributed to the decrease in hospital admissions not only in the telemedical care group but also in the control group. This suggests that the enhanced staff skills and heightened clinical vigilance benefitted all residents of the NH independent of the direct telemedical intervention itself.

Moreover, the enhanced collaboration between nursing staff and the GP should not be underestimated. In addition to adequate staffing, cooperation is considered a key factor in preventing unnecessary hospital admission from NHs, as emphasised by Valk-Draad and Bohnet-Joschko [62]. Close physician-nurse cooperation has also been associated with more rational and needs-oriented resource utilisation and a higher quality of care [63]. The regular communication enabled by the televisits likely fostered mutual trust, improved information exchange, and facilitated coordinated decision-making. The low-threshold accessibility of the GP via telemedicine may thus have promoted a more integrated, team-based approach to care, enabling earlier and more effective interventions and ultimately reducing the need for hospital transfers.

The ripple effects of such a system are considerable. Earlier medical interventions and closer monitoring reduce the likelihood of severe deterioration, thereby relieving pressure on emergency medical services. Fewer emergency calls, fewer ambulance deployments, and fewer hospital admissions also alleviate the strain on overcrowded EDs.

Most importantly, the residents themselves benefit: unnecessary hospital transfers are avoided, and the physical and psychological burdens of unplanned hospitalisations– such as confusion, delirium, or loss of functional abilities– are prevented. Continuity of care by a familiar GP in the trusted NH environment allows for a more person-centered and dignified form of medical care, particularly for frail older adults with complex chronic conditions.

In summary, regular televisits by a GP represent a promising model for improving ambulatory care in NHs. This approach not only reduces unplanned hospital admissions but also strengthens interdisciplinary collaboration, supports nursing staff, alleviates system-level burdens and enhances resident-centered care. Against the backdrop of growing healthcare workforce shortages and rising demands in aging populations, telemedicine emerges as a viable and necessary innovation in long-term care.

### Limitations

There are several limitations to our study. One of these lies in the specific setting of this study. Since it was conducted in a single NH in rural Germany this limits the generalisability of our findings. Adding to this, the limited sample size is another factor to be taken into consideration. Another limitation lies in the retrospective nature of our study. Future studies should be carried out in more than one facility, including a bigger study population and be prospective by design. We did however try to account for these limitations by adding a comparison time period in the past, before televisits were implemented in the NH, to better understand the impact of regular GP televisits in the context of NHs.

A significant limitation is posed by the uncertainty of the potential impact of the COVID-19 pandemic on our findings. It remains unclear if, for our study period (2021/22), the hospitalisations of NH residents were still influenced by the ongoing COVID-19 pandemic, as it had been the case at the beginning of the pandemic, where hospital admissions among NH residents noticeably decreased [64, 65]. This initial decrease has been attributed to changes in care strategies, strict preventive measures, as well as a heightened risk awareness among NH residents, nursing staff and physicians [65–67]. However, in 2021/22, the number of hospital admissions could likely have returned to its pre-pandemic state, due to an ease of the situation in NHs, as a result of vaccinations and the end of the second lockdown in May 2021 [68, 69]. On the contrary, it is also entirely plausible that hospital admissions could even have increased, as a consequence of neglected medical care and missed appointments, which might have led to worsening health problems, especially among chronically ill people [70, 71]. Despite these pandemic-related uncertainties, the observed reduction in unplanned hospital admissions in the telemedical care group - which was significantly lower than in the control group (*P* =.04) - suggests a measurable benefit of regular televisits, independent of broader pandemic effects. Furthermore, the comparison period of 2018/19, which was deliberately selected to precede the onset of the COVID-19 pandemic, helps to contextualise and relativise potential pandemic-related influences on our study outcomes.

Other factors that might have an effect on our study, such as fluctuations in the number and composition of residents (e.g., due to new admissions, discharges, or deaths), staff changes in the group of treating GPs or development and updates of health care standards and documentation processes, could be considered as potential limitations. Nevertheless, the baseline characteristics of residents were comparable for the study periods of 2018/19 and 2021/22. Staff changes in the group of other GPs as well as modifications in standards of care cannot be ruled out. However, the NH was led by the same head of nursing services for the two time periods (2018/19 and 2021/22) and therefore maintained the same nursing care management and standards on site.

With regard to data quality, partially incorrect documentation cannot be entirely ruled out, as the data was collected from the nursing home’s routine care documentation system. However, this documentation is included into the facility’s quality management system and is regularly checked for correctness by the Medical Service of the Health Insurance Companies.

One limitation regarding the interpretation of our findings lies in the inherently limited scope of action available to the treating GP during a televisit. In cases of acute or severe health deteriorations, the televisit may need to be terminated in favour of an unscheduled on-site visit by the GP or the involvement of emergency services. It therefore cannot be ruled out that in individual cases, such follow-up measures - whether an on-site visit by the GP or the support by emergency services - may have contributed to preventing an unplanned hospital admission.

### Strengths

One of the strengths of this study lies in its design, which allowed for a double comparison of the telemedical effect. Besides looking at hospital admissions pre- and post-intervention, namely in the study periods of 2018/19 and 2021/22, the telemedical care group was also compared to a control group which received no televisits. This dual approach strengthens both the internal validity and the reliability of our findings by allowing us to control for temporal trends as well as group-specific effects.

Apart from this, our study benefitted from being part of a research project, which enabled a well-thought out and carefully planned implementation process of the televisits into the NH setting. The project received dedicated funding, which facilitated integration into the daily workflows of both the NH and the GP practice. As a result, certain common barriers to implementing new technology, particularly telemedical systems, in these kinds of settings, could be successfully overcome. These barriers include financial and organisational hurdles - such as the need for initial investments, appropriate reimbursement systems, sufficient telematic infrastructure, compliance with legal requirements - which pose significant obstacles to the adoption but also more broadly to the outcome and sustainability of telemedical implementations [43, 72–77]. Additionally, user-related factors, including acceptance, digital literacy, and perceived competence in using such systems, play a crucial role in the success of telemedical solutions [72, 78]. In the case of our study, most of these potential barriers were addressed through the framework of the funded project. Crucially, short-term financial barriers were removed, the necessary technical infrastructure was already in place, and the acceptance of the used telemedical system had been demonstrated in previous studies.

Most notably, the structured implementation framework within the research project not only supported the practical rollout of the televisits but also strengthened the scientific quality of our study. The availability of dedicated resources enabled a high level of standardisation in training and use, minimised variability in the application of the intervention, and allowed for ongoing monitoring of its implementation. This contributed to a more consistent delivery of the telemedical intervention, enhanced data quality, and clearer attribution of observed effects to the intervention itself. These conditions can be regarded as methodological strengths of our study, increasing both its validity and the reliability of its findings.

## Conclusions

The implementation of regular televisits conducted by the treating GP into routine medical care for NH residents proves to be an effective strategy for reducing unplanned hospital admissions. This effect is likely driven by more frequent medical assessments, enabling early detection and timely management of health deteriorations. Improved collaboration and communication between nursing staff and physicians further strengthen care continuity and responsiveness. Additionally, structured staff training during the study period may have enhanced clinical competence across the board, contributing to improved outcomes even among residents who did not receive televisits. Importantly, reduced hospitalisations spare residents the physical and psychological burdens of acute transfers and protect them from hospital-associated risks. At the same time, the healthcare system benefits from a decreased strain on emergency services and hospitals. Against the backdrop of growing workforce shortages in geriatric care, telemedicine emerges as a sustainable solution that enhances care quality for NH residents, while supporting staff and relieving system pressures.

## Supplementary Information

Supplementary Material 1.

Supplementary Material 2.

Supplementary Material 3.

Supplementary Material 4.

Supplementary Material 5.

Supplementary Material 6.

## Data Availability

All data discussed and analysed in the article are included in this publication and in its supplementary information. The raw datasets used to generate tables and supplementary materials are available from the corresponding author on reasonable request.

## Abbreviations

ACSC: Ambulatory care-sensitive condition
ED: Emergency Department
e.g.: For example
GP: General practitioner
HCP: Health care professional
NH: Nursing home
NHSC: Nursing home-sensitive condition
POC: Point-of-care
&: And
