# Supplementary material for: Impact of regular televisits on unplanned hospital admissions of nursing home residents in rural Germany: a pre-post intervention study
Source: BMC Geriatr. 2025 Sep 8;25:687. doi: 10.1186/s12877-025-06244-6 (PMC12418664; doi:10.1186/s12877-025-06244-6)
Supplement: Supplementary file 1 — Supplementary Material 1. [file 12877_2025_6244_MOESM1_ESM.pdf]

Supplementary Material 1. Baseline characteristics of the resident group of GP1 on the 01.08.2018 and the 01.08.2021: data listed as mean with standard deviation (SD) and counts (n) with percentages (%).

		Missing	01.08.2018	01.08.2021	*P*-Value
**n**			23	23	
**Gender, n (%)**	**Male**	0	6 (26.1)	6 (26.1)	1.000
	**Female**		17 (73.9)	17 (73.9)	
**Age, mean (SD)**		0	85.2 (7.7)	86.0 (8.2)	0.740
**Care level, n (%)**	**2**	0	2 (8.7)	2 (8.7)	0.885
	**3**		4 (17.4)	6 (26.1)	
	**4**		9 (39.1)	7 (30.4)	
	**5**		8 (34.8)	8 (34.8)	
**Length of stay in the NH, mean (SD)**		0	3.5 (3.5)	3.7 (4.0)	0.866
**Number of hospitalisations since moving into the NH, mean (SD)**		0	2.7 (3.1)	3.0 (4.1)	0.807
**Days of hospitalisation since moving into the NH, mean (SD)**		0	22.4 (32.4)	24.7 (32.9)	0.812
**Geriatric syndromes:**					
**Aconuresis, n (%)**		0	7 (30.4)	12 (52.2)	0.231
**Anal incontinence, n (%)**		0	8 (34.8)	9 (39.1)	1.000
**Dementia/Cognitive impairment, n (%)**		0	16 (69.6)	15 (65.2)	1.000
**Fall risk, n (%)**		0	22 (95.7)	21 (91.3)	1.000
**Gait & mobility disorder, n (%)**		0	4 (17.4)	6 (26.1)	0.721
**Immobility, n (%)**		0	2 (8.7)	2 (8.7)	1.000
**Cardiovascular risk factors:**					
**Arterial hypertension, n (%)**		0	16 (69.6)	18 (78.3)	0.737
**Diabetes mellitus, n (%)**		0	3 (13.0)	9 (39.1)	0.093
**Adiposity (BMI≥30), n (%)**		1	5 (21.7)	4 (18.2)	1.000
**Hyperlipidaemia/Dyslipidaemia, n (%)**		0	11 (47.8)	13 (56.5)	0.768
**MARKER ischaemic risk:**					
**CHD/Status post cardiac infarction/Atherosclerosis/Vascular stenosis/(P)AOD, n (%)**		0	10 (43.5)	8 (34.8)	0.763
**MARKER neuropsychiatric risk:**					
**Dementia/Cognitive impairment/Schizophrenic disorder/Psychotic disorder, n (%)**		0	17 (73.9)	15 (65.2)	0.749
**MARKER respiratory risk:**					
**COPD/Bronchial asthma/Chronic bronchitis, n (%)**		0	1 (4.3)	3 (13.0)	0.608
**MARKER gastrointestinal risk:**					
**Gastritis/Oesophagitis/Gastroesophageal reflux, n (%)**		0	6 (26.1)	9 (39.1)	0.529
**Cardiac arrhythmia, n (%)**		0	6 (26.1)	10 (43.5)	0.353
**Cardiac pacemaker, n (%)**		0	2 (8.7)	2 (8.7)	1.000
**Cardiac insufficiency, n (%)**		0	4 (17.4)	5 (21.7)	1.000
**Renal insufficiency, n (%)**		0	3 (13.0)	6 (26.1)	0.459
**Hypotension, n (%)**		0		1 (4.3)	1.000
**Hypothyroidism, n (%)**		0	2 (8.7)		0.489
**Chronic pain, n (%)**		0	3 (13.0)	8 (34.8)	0.167
**Morbus Parkinson/Parkinson syndrome, n (%)**		0	3 (13.0)	1 (4.3)	0.608
**Status post apoplexy/TIA, n (%)**		0	4 (17.4)	4 (17.4)	1.000

Abbreviations. NH: nursing home; BMI: body mass index; CHD: coronary heart disease; (P)AOD: (peripheral) arterial occlusive disease; COPD: chronic obstructive pulmonary disease; TIA: transient ischaemic attack.
