# Supplementary material for: Impact of regular televisits on unplanned hospital admissions of nursing home residents in rural Germany: a pre-post intervention study
Source: BMC Geriatr. 2025 Sep 8;25:687. doi: 10.1186/s12877-025-06244-6 (PMC12418664; doi:10.1186/s12877-025-06244-6)
Supplement: Supplementary file 2 — Supplementary Material 2. [file 12877_2025_6244_MOESM2_ESM.pdf]

Supplementary Material 2. Baseline characteristics of the resident group of GP1 and the resident group of other GPs on the 01.08.2018: data listed as mean with standard deviation (SD) and counts (n) with percentages (%).

		Missing	GP1	Other GPs	*P* -Value
n			23	62	
Gender, n (%)	Male	0	6 (26.1)	15 (24.2)	1.000
	Female		17 (73.9)	47 (75.8)	
Age, mean (SD)		0	85.2 (7.7)	86.4 (6.6)	0.490
Care level, n (%)	2	0	2 (8.7)	8 (12.9)	0.430
	3		4 (17.4)	20 (32.3)	
	4		9 (39.1)	20 (32.3)	
	5		8 (34.8)	14 (22.6)	
Length of stay in the NH, mean (SD)		0	3.5 (3.5)	2.6 (3.2)	0.265
Number of hospitalisations since moving into the NH, mean (SD)		0	2.7 (3.1)	1.5 (1.9)	0.077
Days of hospitalisation since moving into the NH, mean (SD)		0	22.4 (32.4)	14.1 (20.6)	0.263
Geriatric syndromes:					
Aconuresis, n (%)		0	7 (30.4)	24 (38.7)	0.652
Anal incontinence, n (%)		0	8 (34.8)	20 (32.3)	1.000
Dementia/Cognitive impairment, n (%)		0	16 (69.6)	32 (51.6)	0.216
Fall risk, n (%)		1	22 (95.7)	53 (85.5)	0.274
Gait & mobility disorder, n (%)		0	4 (17.4)	11 (17.7)	1.000
Immobility, n (%)		0	2 (8.7)	4 (6.5)	0.660
Cardiovascular risk factors:					
Arterial hypertension, n (%)		0	16 (69.6)	42 (67.7)	1.000
Diabetes mellitus, n (%)		0	3 (13.0)	19 (30.6)	0.172
Adiposity (BMI≥30), n (%)		1	5 (21.7)	11 (17.7)	0.757
Hyperlipidaemia/Dyslipidaemia, n (%)		0	11 (47.8)	33 (53.2)	0.843
MARKER ischaemic risk:					
CHD/Status post cardiac infarction/Atherosclerosis/Vascular stenosis/(P)AOD, n (%)		0	10 (43.5)	29 (46.8)	0.979
MARKER neuropsychiatric risk:					
Dementia/Cognitive impairment/Schizophrenic disorder/Psychotic disorder, n (%)		0	17 (73.9)	33 (53.2)	0.141
MARKER respiratory risk:					
COPD/Bronchial asthma/Chronic bronchitis, n (%)		0	1 (4.3)	6 (9.7)	0.668
MARKER gastrointestinal risk:					
Gastritis/Oesophagitis/Gastroesophageal reflux, n (%)		0	6 (26.1)	15 (24.2)	1.000
Cardiac arrhythmia, n (%)		0	6 (26.1)	23 (37.1)	0.488
Cardiac pacemaker, n (%)		0	2 (8.7)	7 (11.3)	1.000
Cardiac insufficiency, n (%)		0	4 (17.4)	9 (14.5)	0.742
Renal insufficiency, n (%)		0	3 (13.0)	9 (14.5)	1.000
Hypotension, n (%)		0		1 (1.6)	1.000
Hypothyroidism, n (%)		0	2 (8.7)	6 (9.7)	1.000
Chronic pain, n (%)		0	3 (13.0)	8 (12.9)	1.000
Morbus Parkinson/Parkinson syndrome, n (%)		0	3 (13.0)	7 (11.3)	1.000
Status post apoplexy/TIA, n (%)		0	4 (17.4)	19 (30.6)	0.344

Abbreviations. NH: nursing home; BMI: body mass index; CHD: coronary heart disease; (P)AOD: (peripheral) arterial occlusive disease; COPD: chronic obstructive pulmonary disease; TIA: transient ischaemic attack.
