# Supplementary material for: Impact of regular televisits on unplanned hospital admissions of nursing home residents in rural Germany: a pre-post intervention study
Source: BMC Geriatr. 2025 Sep 8;25:687. doi: 10.1186/s12877-025-06244-6 (PMC12418664; doi:10.1186/s12877-025-06244-6)
Supplement: Supplementary file 3 — Supplementary Material 3. [file 12877_2025_6244_MOESM3_ESM.pdf]

Supplementary Material 3. Hospitalisation causes of the resident group of GP1 and the resident group of other GPs in 2018/19: data listed as counts (n) with percentages (%).

	Missing	Overall	GP1	Other GPs	*P* -Value
**n**		74	19	55	
**Breathing difficulties/Dyspnoea, n (%)**	0	7 (9.5)		7 (12.7)	0.180
**Gastrointestinal problems, n (%)**	0	7 (9.5)	1 (5.3)	6 (10.9)	0.669
**Cardiovascular disorder, n (%)**	0	3 (4.1)		3 (5.5)	0.565
**Fall, n (%)**	0	19 (25.7)	4 (21.1)	15 (27.3)	0.764
**General health status deterioration (unclear genesis), n (%)**	0	12 (16.2)	4 (21.1)	8 (14.5)	0.491
**Urologic problems, n (%)**	0	1 (1.4)	1 (5.3)		0.257
**Nephrological problems, n (%)**	0	2 (2.7)		2 (3.6)	1.000
**Thoracic pain, n (%)**	0	5 (6.8)	2 (10.5)	3 (5.5)	0.598
**Abdominal pain, n (%)**	0	1 (1.4)		1 (1.8)	1.000
**Suspected apoplexy/TIA, n (%)**	0	5 (6.8)	2 (10.5)	3 (5.5)	0.598
**Suspected pneumonia, n (%)**	0	3 (4.1)		3 (5.5)	0.565
**Psychopathological anomaly, n (%)**	0	3 (4.1)		3 (5.5)	0.565
**Suspected thrombosis, n (%)**	0	2 (2.7)		2 (3.6)	1.000
**Oedema, n (%)**	0	2 (2.7)		2 (3.6)	1.000
**Inflammation signs of the lower extremities, n (%)**	0	3 (4.1)	1 (5.3)	2 (3.6)	1.000
**Exsiccosis n (%)**	0	1 (1.4)	1 (5.3)		0.257
**Dermatological anomaly, n (%)**	0	1 (1.4)	1 (5.3)		0.257
**Medication adjustment, n (%)**	0	1 (1.4)	1 (5.3)		0.257
**Suspected lung embolism, n (%)**	0	1 (1.4)	1 (5.3)		0.257

Abbreviation. TIA: transient ischaemic attack.
