# Supplementary material for: Impact of regular televisits on unplanned hospital admissions of nursing home residents in rural Germany: a pre-post intervention study
Source: BMC Geriatr. 2025 Sep 8;25:687. doi: 10.1186/s12877-025-06244-6 (PMC12418664; doi:10.1186/s12877-025-06244-6)
Supplement: Supplementary file 4 — Supplementary Material 4. [file 12877_2025_6244_MOESM4_ESM.pdf]

Supplementary Material 4. Hospitalisation causes of all admissions in 2018/19 and 2021/22: data listed as counts (n) with percentages (%).

	Missing	2018/19	2021/22	*P* -Value
**n**		74	55	
**Breathing difficulties/Dyspnoea, n (%)**	3	7 (9.5)	7 (13.5)	0.678
**Gastrointestinal problems, n (%)**	3	7 (9.5)	5 (9.6)	1.000
**Seizure, n (%)**	3		2 (3.8)	0.168
**Fall, n (%)**	3	19 (25.7)	18 (34.6)	0.376
**General health status deterioration (unclear genesis), n (%)**	3	12 (16.2)	10 (19.2)	0.841
**Urological problems, n (%)**	3	1 (1.4)	4 (7.7)	0.159
**Pain of the lower extremities, n (%)**	3		2 (3.8)	0.168
**Thoracic pain, n (%)**	3	5 (6.8)	5 (9.6)	0.740
**Abdominal pain, n (%)**	3	1 (1.4)	1 (1.9)	1.000
**Hemic anomaly, n (%)**	3		1 (1.9)	0.413
**Syncope, n (%)**	3		1 (1.9)	0.413
**Cardiovascular disorder, n (%)**	3	3 (4.1)		0.267
**Nephrological problems, n (%)**	3	2 (2.7)		0.511
**Suspected apoplexy/TIA, n (%)**	3	5 (6.8)		0.077
**Suspected pneumonia, n (%)**	3	3 (4.1)		0.267
**Psychopathological anomaly, n (%)**	3	3 (4.1)		0.267
**Suspected thrombosis, n (%)**	3	2 (2.7)		0.511
**Oedema, n (%)**	3	2 (2.7)		0.511
**Inflammation signs of the lower extremities, n (%)**	3	3 (4.1)		0.267
**Exsiccosis, n (%)**	3	1 (1.4)		1.000
**Dermatological anomaly, n (%)**	3	1 (1.4)		1.000
**Medication adjustment, n (%)**	3	1 (1.4)		1.000
**Suspected lung embolism, n (%)**	3	1 (1.4)		1.000

Abbreviation. TIA: transient ischaemic attack.
