# Supplementary material for: Impact of regular televisits on unplanned hospital admissions of nursing home residents in rural Germany: a pre-post intervention study
Source: BMC Geriatr. 2025 Sep 8;25:687. doi: 10.1186/s12877-025-06244-6 (PMC12418664; doi:10.1186/s12877-025-06244-6)
Supplement: Supplementary file 5 — Supplementary Material 5. [file 12877_2025_6244_MOESM5_ESM.pdf]

Supplementary Material 5. Hospitalisation causes of the telemedical care group and the control group in 2021/22: data listed as counts (n) with percentages (%).

	Missing	Overall	Telemedical care group	Control group	*P* -Value
**n**		55	15	40	
**Breathing difficulties/Dyspnoea, n (%)**	3	7 (13.5)	3 (20.0)	4 (10.8)	0.397
**Gastrointestinal problems, n (%)**	3	5 (9.6)		5 (13.5)	0.305
**Seizure, n (%)**	3	2 (3.8)	1 (6.7)	1 (2.7)	0.498
**Fall, n (%)**	3	18 (34.6)	8 (53.3)	10 (27.0)	0.138
**General health status deterioration (unclear genesis), n (%)**	3	10 (19.2)		10 (27.0)	0.046
**Urologic problems, n (%)**	3	4 (7.7)		4 (10.8)	0.311
**Pain of the lower extremities, n (%)**	3	2 (3.8)		2 (5.4)	1.000
**Thoracic pain, n (%)**	3	5 (9.6)	2 (13.3)	3 (8.1)	0.619
**Abdominal pain, n (%)**	3	1 (1.9)	1 (6.7)		0.288
**Hemic anomaly, n (%)**	3	1 (1.9)	1 (6.7)		0.288
**Syncope, n (%)**	3	1 (1.9)	1 (6.7)		0.288
