# Supplementary material for: Impact of regular televisits on unplanned hospital admissions of nursing home residents in rural Germany: a pre-post intervention study
Source: BMC Geriatr. 2025 Sep 8;25:687. doi: 10.1186/s12877-025-06244-6 (PMC12418664; doi:10.1186/s12877-025-06244-6)
Supplement: Supplementary file 6 — Supplementary Material 6. [file 12877_2025_6244_MOESM6_ESM.docx]

**Supplementary Material 6.** Reporting guideline based on the STROBE Statement.

| \|  \| Item \| Recommendation \| Page(s) \| \| --- \| --- \| --- \| --- \| \| **Title and abstract** \| 1 \| (*a*) Indication of the study’s design with a commonly used term in the title or the abstract \| 1 \| \| (*b*) Provision of an informative and balanced summary of what was done and what was found in the abstract \| 2,3 \| \| Introduction \| \| \|  \| \| Background/rationale \| 2 \| Explanation of the scientific background and rationale for the investigation being reported \| 3-6 \| \| Objectives \| 3 \| Statement of specific objectives, including any prespecified hypotheses \| 6 \| \| Methods \| \| \|  \| \| Study design \| 4 \| Presentation of key elements of study design early in the paper \| 6,7 \| \| Setting \| 5 \| Description of the setting, locations, and relevant dates, including periods of recruitment, exposure, follow-up, and data collection \| 7,8 \| \| Participants \| 6 \| Eligibility criteria, and the sources and methods of selection of participants. Description of follow-up methods \| 7 \| \| Variables \| 7 \| Clear definition of all outcomes, exposures, predictors, potential confounders, and effect modifiers. \| 7-10 \| \| Data sources/ measurement \| 8 \| For each variable of interest, giving sources of data and details of methods of assessment (measurement). Description of comparability of assessment methods if there is more than one group \| 10,11 \| \| Bias \| 9 \| Description of any efforts to address potential sources of bias \| 10,11 \| \| Study size \| 10 \| Explanation on how the study size was arrived at \| 6,7 \| \| Quantitative variables \| 11 \| Explanation on how quantitative variables were handled in the analyses. If applicable, describe which groupings were chosen and why \| 13 \| \| Statistical methods \| 12 \| (*a*) Description of all statistical methods, including those used to control for confounding \| 13,14 \| \| (*b*) Description of any methods used to examine subgroups and interactions \| 13,14 \| \| (*c*) Explanation on how missing data were addressed \| 14 \| \| Results \| \| \|  \| \| Participants \| 13 \| Reporting of numbers of individuals at each stage of study—eg numbers potentially eligible, examined for eligibility, confirmed eligible, included in the study, completing follow-up, and analysed \| 14,15 \| \| Descriptive data \| 14 \| (a) Giving characteristics of study participants (eg demographic, clinical, social) and information on exposures and potential confounders \| 14,15 \| \| (b) Indication of number of participants with missing data for each variable of interest \| 14,15 \| \| (c) Summarising of follow-up time (eg, average and total amount) \| 15 \| \| Outcome data \| 15 \| Reporting of numbers of outcome events or summary measures over time \| 15-17 \| \| Main results \| 16 \| Giving unadjusted estimates and, if applicable, confounder-adjusted estimates and their precision (eg, 95% confidence interval). Making clear which confounders were adjusted for and why they were included \| 15-17 \| \| Other analyses \| 17 \| Reporting of other analyses done—eg analyses of subgroups and interactions, and sensitivity analyses \| 17 \| \| Discussion \| \| \|  \| \| Key results \| 18 \| Summarising key results with reference to study objectives \| 17,18 \| \| Limitations \| 19 \| Discussion of limitations of the study, taking into account sources of potential bias or imprecision. Discussion of both direction and magnitude of any potential bias \| 21,22 \| \| Interpretation \| 20 \| Giving a cautious overall interpretation of results considering objectives, limitations, multiplicity of analyses, results from similar studies, and other relevant evidence \| 18-20 \| \| Generalisability \| 21 \| Discussion of the generalisability (external validity) of the study results \| 21 \| \| Other information \| \| \|  \| \| Funding \| 22 \| Giving the source of funding and the role of the funders for the present study and, if applicable, for the original study on which the present article is based \| 24 \| |
| --- | --- | --- | --- | --- | --- | --- | --- | --- | --- | --- | --- | --- | --- | --- | --- | --- | --- | --- | --- | --- | --- | --- | --- | --- | --- | --- | --- | --- | --- | --- | --- | --- | --- | --- | --- | --- | --- | --- | --- | --- | --- | --- | --- | --- | --- | --- | --- | --- | --- | --- | --- | --- | --- | --- | --- | --- | --- | --- | --- | --- | --- | --- | --- | --- | --- | --- | --- | --- | --- | --- | --- | --- | --- | --- | --- | --- | --- | --- | --- | --- | --- | --- | --- | --- | --- | --- | --- | --- | --- | --- | --- | --- | --- | --- | --- | --- | --- | --- | --- | --- | --- | --- | --- | --- | --- | --- | --- | --- | --- | --- | --- | --- | --- | --- | --- | --- | --- | --- | --- | --- | --- | --- |
